# Supplementary material for: Passive frequency comb generation at radiofrequency for ranging applications
Source: Nat Commun. 2024 Apr 2;15:2844. doi: 10.1038/s41467-024-46940-2 (PMC10987526; doi:10.1038/s41467-024-46940-2)
Supplement: Supplementary file 1 — Supplementary Information [file 41467_2024_46940_MOESM1_ESM.docx]

**Supplementary Information for**

**Passive frequency comb generation at radiofrequency for ranging applications**

Hussein M. E. Hussein^1,2*^, Seunghwi Kim^3,*^, Matteo Rinaldi^1,2^, Andrea Alù^3,4,†^ and Cristian Cassella^1,2,†^

^1^Department of Electrical and Computer Engineering, Northeastern University, Boston, MA, USA

^2^Institute of NanoSystems Innovation, Boston, MA, USA

^3^Photonics Initiative, Advanced Science Research Center, City University of New York, New York, New York 10031, USA

^4^Physics Program, Graduate Center, City University of New York, New York, NY 10016, USA

^†^Corresponding author: [c.cassella@northeastern.edu](mailto:c.cassella@northeastern.edu) and [aalu@gc.cuny.edu](mailto:aalu@gc.cuny.edu)

^*^These authors contributed equally to this work

Supplementary Table 1: Table of Symbols

| **Symbol** | **Definition** |
| --- | --- |
| $P_{th}$ | Threshold power for frequency comb generation |
| $P_{r}$ | Power received by the qHT |
| $d$ | Distance between the qHT and the interrogator |
| $\Delta f$ | Comb line spacing |
| $\Delta f_{\max}$ | Maximum comb line spacing at $P_{r}$=$P_{th}$ |
| $a_{1}, a_{2}$ | Normalized amplitudes of the signal and pump modes |
| $b$ | Normalized amplitude of the mechanical mode in the SAW resonator |
| $\omega_{p}$ | Angular frequency of the interrogation signal (pump signal) $\omega_{p}=2\pi f_{p}$ |
| $\omega_{1}, \omega_{2}$ | Angular frequencies of the signal and pump modes, i.e., $\omega_{1}=2\pi f_{1}$ and $\omega_{2}=2\pi f_{2}$are the resonant frequencies of LC tanks in the input and output meshes |
| $\omega_{b}$ | Resonant frequency of the mechanical mode, i.e., the resonant frequency of the SAW device is $\omega_{b}=2\pi f_{b}$ |
| $\kappa_{1}, \kappa_{2}$ | Loss rates of the electrical modes $a_{1}$ and $a_{2}$. Here $\kappa\equiv\kappa_{1}$ is used throughout the main text. |
| $\kappa_{ex}$ | External coupling rate to the pump mode $a_{1}$ |
| $\Gamma$ | Loss rate of the mechanical mode $b$ |
| $Q$ | Quality factor of the mechanical modes $Q=\omega_{b}/\Gamma$ |
| $\Delta_{a}$ | Detuning from half the pump frequency to the signal mode $a_{1}$ $\Delta_{a}=\omega_{a}-\omega_{p}/2$ |
| $\Delta_{b}$ | Detuning from half the pump frequency to the mechanical mode $b$ $\Delta_{b}=\omega_{b}-\omega_{p}/2$ |
| $G$ | Electromechanical coupling rate |
| $g$ | Coupling rate of the second-order nonlinearity $\chi^{\left(2\right)}$ |
| $r_{1}$ | Gain saturation coefficient |
| $r_{2}$ | Small signal gain parameter |
| TX/RX | Transmitter/Receiver of interrogation node |
| A1 | Directive Yagi antenna to generate a CW interrogation signal at $\omega_{p}$ |
| A2 | Dipole antenna to wirelessly receive a portion of the backscattered signal generated by the qHT |

# Derivation of the model presented in the main manuscript

In this section, we thoroughly study the model presented in the main text and we show its complete derivation. Supplementary Supplementary Figure 1 illustrates the schematic of the system under investigation, consisting of a double-resonant system mediated by the $\chi^{\left(2\right)}$ nonlinearity [1]. The system can be described by the temporal coupled-mode theory as follows:

Here, $a_{i}$ is the amplitude of the intracavity fields of the two modes, $\kappa_{i}$and $\omega_{i}$ are the corresponding total loss rate and resonant frequency. $a_{in}$ is the external pump, $\omega_{p}$is the pump frequency and$\kappa_{ex}$is the external coupling rate from the pump to the resonant mode. *g* is the nonlinear coefficient representing the $\chi_{2}$ process, giving the self and cross-phase modulation effects of the two modes. Assuming the pump mode (*a*_2_) is broadband compared to the signal mode ($a_{1}$), i.e., $\kappa_{2}>\kappa_{1}$, we can simplify Eq. by adiabatically eliminating the pump and choosing a frame rotating with the half frequency of the pump $a\to a^{-i\omega_{p}/2t}$:

where $r_{1}\approx\frac{2\left|g\right|^{2}}{\kappa_{2}}$is the gain saturation coefficient,$r_{2}\approx\frac{2g\sqrt{\kappa_{ex}}a_{in}}{\kappa_{2}}$ is the small-signal gain parameter, and the detuning is given by$\Delta_{a}=\omega_{a}-\frac{\omega_{p}}{2}$. Note that an input field is normalized so that the input pump power () is identical to the qHT’s received power (i.e., $P_{r}\equiv P_{in}$). Therefore, the small-signal gain parameter can be written as . Here, for improved clarity we drop the subscription 1 since only *a*_1_ will be considered from now on.

Now, we include the coupling of *a* to the mechanical mode as described in the main text.

Here, *b* is the amplitude of the intracavity field of the mechanical mode, $\Gamma$ is the mechanical loss rate, and *G* is the electromechanical coupling rate. $\Delta_{b}=\omega_{b}-\frac{\omega_{p}}{2}$is the mechanical detuning parameter with respect to half the frequency of the pump due to the change of the frame for the *b* mode$b\to b^{-i\omega_{p}/2t}$, where $\omega_{b}$ is the mechanical resonant frequency. The relationship among all frequencies is visually represented in Supplementary Fig. 1 A and B.


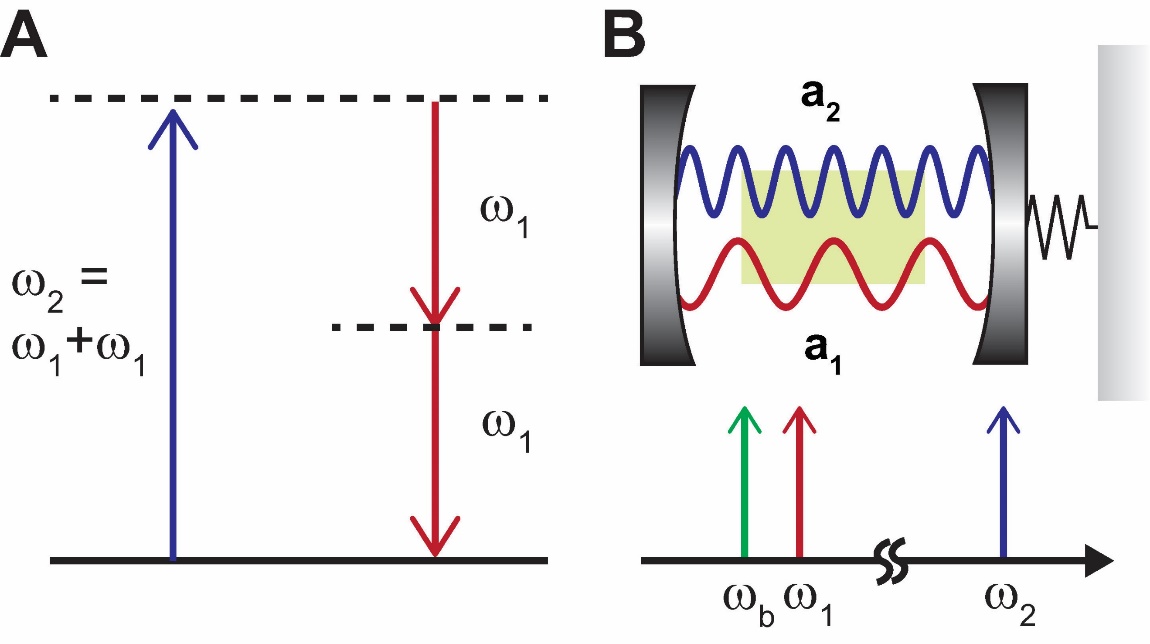


Supplementary Figure 1 A Schematic of the virtual energy levels for the $\chi^{\left(2\right)}$ process. For the doubly resonant systems, the modes $a_{1}$ and $a_{2}$ are resonant at $\omega_{1}$ and $\omega_{2}$ respectively, and they are depicted as the virtual energy-level description. B Schematic of the system of interest: $a_{1}$ is parametrically amplified by $a_{2}$, while being coupled to *b*. *b* has a natural resonant frequency denoted as $\omega_{b}$.

# Detailed analysis of the system

In the main text, we studied our systems in the two cases: 1) the weak coupling case, $G\ll\Gamma/2$, and 2) the strong coupling case, $G>\Gamma/2$. Here, we thoroughly study these two cases by employing the linear stability analysis. The two equations of motion in Eq. are simplified to a single equation by solving for *a*:

where $f\left(\tau\right)\equiv e^{-\left(\frac{\Gamma}{2}+i\Delta_{b}\right)\tau}$. The last term is non-Markovian.

## Weak coupling case

$a\left(t-\tau\right)$ is considered $a\left(t\right)$ due to the Markovian approximation [2]. Thus, Eq. is simplified by:

with the effective loss rate and detuning, $\bar{\kappa}=\kappa+\frac{G^{2}}{\left(\Gamma/2\right)^{2}+\Delta_{b}^{2}}\Gamma$and $\bar{\Delta}_{a}=\Delta_{a}-\frac{G^{2}}{\left(\Gamma/2\right)^{2}+\Delta_{b}^{2}}\Delta_{b}$. By assuming *a*(*t*) to reach its steady state, we get the quadratic relation from Eq. .

$$r_{1}^{2}X^{3}+r_{1}\overline{\kappa}X^{2}+\left[\left(\frac{\overline{\kappa}}{2}\right)^{2}+\overline{\Delta}_{a}^{2}-r_{2}^{2}\right]X=0,$$

where $X=\left|a\right|^{2}$. We have three possible solutions for Eq. , which are:

$$\begin{matrix} X=0 , \\ X=-\frac{\overline{\kappa}}{2r_{1}}\pm\sqrt{r_{2}^{2}-\overline{\Delta}_{a}^{2}}. \end{matrix}$$

Here, the nonzero solution of Eq. is available when the gain parameter is larger than its critical value, i.e., $r_{2}\geq r_{2.cr}^{weak}$ where $r_{2.cr}^{weak}=\sqrt{\overline{\Delta}_{a}^{2}+\left(\kappa/2+2G^{2}/\Gamma\right)^{2}}$. However, we are not sure whether the weak coupling scenario can provide comb-spectra even under enough $r_{2}$. Therefore, we should linearize Eq. and monitor its stability by using the linear stability analysis. To linearize the system, we apply the first order of the perturbative series, $a\left(t\right)=\alpha+\delta a\left(t\right)$ to Eq. .

$$\frac{d\delta a}{dt}=-\left(\frac{\overline{\kappa}}{2}+i\overline{\Delta}_{a}\right)\delta a-r_{1}\left[2\delta a\left|\alpha\right|^{2}+\delta a^{*}\alpha^{2}\right]-ir_{2}\delta a^{*}.$$

The Laplace transform applies to such a linearized equation in Eq. by defining $\delta a\left(s\right)=\int_{0}^{\infty} dt\delta a\left(t\right)e^{-st}$ and $\delta a^{*}\left(s\right)=\int_{0}^{\infty} dt\delta a^{*}\left(t\right)e^{-st}$. Then, Eq. is rewritten in its matricial representation, $MV\left(s\right)=V\left(0\right)$ where $V\left(s\right)^{T}=\left[\delta a\left(s\right), \delta a^{*}\left(s\right)\right]^{T}$ . In order to monitor the stability of the system, we calculate the poles of Eq. by searching for the *s* values that null the determinant of M.

$$\left(s+\frac{\overline{\kappa}}{2}+i\overline{\Delta}_{a}+2r_{1}\left|\alpha\right|^{2}\right)\left(s+\frac{\overline{\kappa}}{2}-i\overline{\Delta}_{a}+2r_{1}\left|\alpha\right|^{2}\right)=r_{1}^{2}\left|\alpha\right|^{4}+r_{2}^{2}.$$

Note that the imaginary part of $\alpha$can be nulled by just shifting the rotating frame. This allows to simplify Eq. and obtain a solution for s as follows:

$$s=-\frac{\overline{\kappa}}{2}-2r_{1}\left|\alpha\right|^{2}\pm\sqrt{r_{1}^{2}\left|\alpha\right|^{4}+r_{2}-\overline{\Delta}_{a}^{2}}.$$

When the nonlinearities are small, i.e., $r_{1}^{2}\left|\alpha\right|^{4}+r_{2}\leq\overline{\Delta}_{a}^{2}$ and $\Delta_{b}\simeq0$, the real and imaginary parts of s are written as:

$$\begin{matrix} \mathrm{Re}\left[s\right]=-\frac{\overline{\kappa}}{2}-2r_{1}\left|\alpha\right|^{2}, \\ \mathrm{Im}\left[s\right]=\pm\sqrt{\overline{\Delta}_{a}^{2}-\left(r_{1}^{2}\left|\alpha\right|^{4}+r_{2}\right)^{2}}\approx\pm\Delta_{a}. \end{matrix}$$

As a result, under the assumption of small nonlinearities our system is always stable and oscillates at the resonant frequency of *a*, $\omega_{a}$. In contrast, for large nonlinearities, we can analyze the system as follows:

$$\begin{matrix} \mathrm{Re}\left[s\right]=-\frac{\overline{\kappa}}{2}-2r_{1}\left|\alpha\right|^{2}\pm\sqrt{\left(r_{1}^{2}\left|\alpha\right|^{4}+r_{2}\right)^{2}-\overline{\Delta}_{a}^{2}}, \\ \mathrm{Im}\left[s\right]=0. \end{matrix}$$

Particularly, the system oscillates at half the frequency of the pump, $\omega_{p}/2$, implying that we cannot see any frequency comb under the weak coupling regime. We note that when $r_{2}$ is larger than its critical value, $r_{2}\geq r_{2.cr}^{\text{weak}}$, the system is not always stable as the real part of *s* can be positive, $\mathrm{Re}\left[s\right]\geq0$.

Supplementary Supplementary Figure 2A shows the phase diagram in the $r_{2}$ and Δ_a_ space for the weak coupling scenario with $\kappa=5\Gamma$, $G=0.1\Gamma$, $\omega_{a}=50\kappa$, $\omega_{b}=49.5\Gamma$ and $r_{1}=0.1\Gamma$. Here,$\omega_{b}$ is considered to be equal to half of the pump frequency, i.e.,$\Delta_{b}=0$. The solid blue line represents the relationship between the critical value of $r_{2}$ and the detuning of the mode *a*. Hence, any states below the blue line are stable since the real part of the pole is smaller than zero (i.e., $\mathrm{Re}\left[s\right]<0$). Likewise, states above the line are unstable due to $\mathrm{Re}\left[s\right]>0$. Supplementary Supplementary Figure 2B-G illustrate the spectrum of the mechanical mode $\left|b\left(\omega/2\pi\right)\right|$ computed via the fast Fourier transform when assuming different $r_{2}$values. As expected in Eq. and , the mechanical mode below the threshold in Supplementary Supplementary Figure 2B oscillates at $\omega_{a}$, while it oscillates at $\omega_{b}$ after the threshold (see Supplementary Supplementary Figure 2C-G). Here, we cannot observe any comb-like spectra, even in the unstable regime that assumes strong nonlinearities.

## Strong coupling case

In the main text, we demonstrated that the comb-like spectra were observable for the large coupling case ($G>\Gamma/2$) and for large $r_{2}$ values, i.e., $P_{\mathrm{r}}>P_{th}$. Here we prove the results shown in the main text and further analyze the system under the strong coupling scenario. Similarly to our previous analysis, we linearize Eq. with the first-order perturbative series.

After applying the Laplace transform into Eq. , we get the condition for the poles[[1]](#footnote-2).

$$s=-\frac{\kappa}{2}-\frac{G^{2}}{s+\Gamma/2}-2r_{1}\left|\alpha\right|^{2}\pm\sqrt{r_{1}^{2}\left|\alpha\right|^{4}+r_{2}-\Delta_{a}^{2}}.$$

When assuming low nonlinearities, the real and imaginary parts of the pole are given by:

$$\begin{matrix} \mathrm{Re}\left[s\right]=-\frac{\kappa}{2}-\frac{G^{2}\left(\mathrm{Re}\left[s\right]+\Gamma/2\right)}{\left(\mathrm{Re}\left[s\right]+\Gamma/2\right)^{2}+\mathrm{Im}\left[s\right]^{2}}-2r_{1}\left|\alpha\right|^{2}, \\ \mathrm{Im}\left[s\right]=\frac{G^{2}\mathrm{Im}\left[s\right]}{\left(\mathrm{Re}\left[s\right]+\Gamma/2\right)^{2}+\mathrm{Im}\left[s\right]^{2}}\pm\sqrt{\Delta_{a}^{2}-\left(r_{1}^{2}\left|\alpha\right|^{4}+r_{2}^{2}\right)}. \end{matrix}$$

Instead, for strong nonlinearities, the real and imaginary parts of the pole are given by:

$$\begin{matrix} \mathrm{Re}\left[s\right]=-\frac{\kappa}{2}-\frac{G^{2}\left(\mathrm{Re}\left[s\right]+\Gamma/2\right)}{\left(\mathrm{Re}\left[s\right]+\Gamma/2\right)^{2}+\mathrm{Im}\left[s\right]^{2}}-2r_{1}\left|\alpha\right|^{2}\pm\sqrt{\left(r_{1}^{2}\left|\alpha\right|^{4}+r_{2}^{2}\right)-\Delta_{a}^{2}}, \\ \mathrm{Im}\left[s\right]=\frac{G^{2}\mathrm{Im}\left[s\right]}{\left(\mathrm{Re}\left[s\right]+\Gamma/2\right)^{2}+\mathrm{Im}\left[s\right]^{2}}. \end{matrix}$$

The solutions of the imaginary part are $\mathrm{Im}\left[s\right]=0$or $\mathrm{Im}\left[s\right]=\pm\sqrt{G^{2}-\left(\mathrm{Re}\left[s\right]+\Gamma/2\right)^{2}}$. It means that we may obtain nonzero $\mathrm{Im}\left[s\right]$ for the unstable solution $\mathrm{Re}\left[s\right]>0$ under the strong coupling condition $G>\Gamma/2$. We plug the nonzero solution of $\mathrm{Im}\left[s\right]$into the equation for $\mathrm{Re}\left[s\right]$ and rewrite the relations:

$$\begin{matrix} \mathrm{Re}\left[s\right]=-\frac{1}{4}\left(\kappa+\Gamma\right)-r_{1}\left|\alpha\right|^{2}\pm\frac{1}{2}\sqrt{\left(r_{1}^{2}\left|\alpha\right|^{4}+r_{2}^{2}\right)-\Delta_{a}^{2}}, \\ \mathrm{Im}\left[s\right]=\pm\sqrt{G^{2}-\left(\mathrm{Re}\left[s\right]+\Gamma/2\right)^{2}}. \end{matrix}$$

As discussed in the main paper, the comb spacing exhibited by our system decreases with the pump power. In fact, the comb spacing is related to the imaginary part of the poles in Eq. . Here, we see that the real part of the poles in Eq. is negative at the beginning under the moderate gain parameter $r_{2}$ and increases with $r_{2}$. Eventually, when $\mathrm{Re}\left[s\right]$becomes zero, it gives the comb spacing $\Delta f_{0}=2\sqrt{G^{2}-\left(\Gamma/2\right)^{2}}$ at the threshold of the instability condition. Note that this formula for the comb spacing is only valid at $\mathrm{Re}\left[s\right]=0$, as the linear stability analysis is no longer valid when Re[s]$>$0.

We also get the condition when the system is unstable, i.e., $\mathrm{Re}\left[s\right]>0$, and it is given by $\left(r_{1}^{2}\left|\alpha\right|^{4}+r_{2}^{2}\right)-\Delta_{a}^{2}>\frac{1}{4}\left(\kappa+\Gamma\right)^{2}+2\left(\kappa+\Gamma\right)r_{1}\left|\alpha\right|^{2}+4r_{1}^{2}\left|\alpha\right|^{4}.$ After some simplifications, this can be rewritten as:

$$3r_{1}^{2}\left|\alpha\right|^{4}+2\left(\kappa+\Gamma\right)r_{1}\left|\alpha\right|^{2}+\frac{1}{4}\left(\kappa+\Gamma\right)^{2}-r_{2}^{2}+\Delta_{a}^{2}<0.$$

Such a quadratic equation in $\left|\alpha\right|^{2}$provides the valid range $\left|\alpha_{-.th}\right|^{2}<\left|\alpha\right|^{2}<\left|\alpha_{+,th}\right|^{2}$, where $r_{1}\left|\alpha_{\pm,th}\right|^{2}=-\frac{1}{3}\left(\kappa+\Gamma\right)\pm\frac{1}{6}\sqrt{12\left(r_{2}^{2}-\Delta_{a}^{2}\right)+\left(\kappa+\Gamma\right)^{2}}$. We have the critical small-signal gain parameter when the square root term is real:

$$r_{2.crit}=\sqrt{\Delta_{a}^{2}+\frac{1}{3}\left(\kappa+\Gamma\right)^{2}}$$

We note that $r_{2.crit}$ is written as a function of the power threshold for frequency comb generation, . Supplementary Supplementary Figure 3A shows the phase diagram in the $r_{2}$ and Δ_a_ space for the strong coupling scenario with $\kappa=5\Gamma$, $G=5\Gamma$, $\omega_{a}=100\kappa$, $\omega_{b}=98\Gamma$ and $r_{1}=0.1\Gamma$. Here half of the pump frequency is at $\omega_{b}$, i.e.,$\Delta_{b}=0$. Unlike the weak coupling case shown in Supplementary Supplementary Figure 3, the spectrum of the mechanical mode *b* shows comb-like responses when $r_{2}\geq r_{2.crit}$. In Supplementary Supplementary Figure 3C, the comb spectrum occurs at $r_{2}$ = $r_{2}$_.crit_, and its comb spacing is identical to $\Delta f_{0}=2\sqrt{G^{2}-\left(\Gamma/2\right)^{2}}$as discussed in the main text. Supplementary Supplementary Figure 4A illustrates the frequency of the two closest peaks in comb spectra as a function of $r_{2}$, and we observe that the comb spacing decreases with $r_{2}$. In the end, combs suddenly disappear, and the spectrum merely shows a single frequency oscillating at $\omega_{p}/2$ as shown in Supplementary Supplementary Figure 4B.

## Intuitive interpretation of comb generation with synchronization aspect

As previously discussed, our prediction is that comb spacing decreases with *r*_2_ or pump power, as illustrated in Supplementary Supplementary Figure 4A, which is experimentally observed in Supplementary Fig. 8A-B. Here, we aim to provide a more qualitative explanation of the observed phenomena.

In Eq. , it is evident that the *a* mode bears resemblance to the well-known Stuart-Landau equation as follows:

Assuming *l* in Eq. is real and positive, $A\left(t\right)$ of the Stuart-Landau oscillator exhibits a stable focus on the phase plane for $\mathrm{Re}\left[\sigma\right]<0$, while it represent a stable limit cycle for $\mathrm{Re}\left[\sigma\right]>0$, having the limiting amplitude as $\left|A\right|\to\sqrt{\frac{2\mathrm{Re}\left[\sigma\right]}{l}}$. Hence, the Stuart-Landau oscillators intrinsically undergo a supercritical bifurcation, signifying two stable points for $\mathrm{Re}\left[\sigma\right]>0$. In our case, the *a*_1_, mode without coupling to the *b* mode in Eq. , has a similar fixed point obtained by solving it with and the stable point is given by $\alpha_{stable}=\sqrt{\frac{\pm r_{2}-\kappa/2}{r_{1}}}$ . This suggests that our equation can exhibit either a supercritical or subcritical bifurcation, depending on the phase of the *a* mode even for $r_{2}\geq\kappa/2$.

It is interesting to note that the dynamics of multiple N interacting Landau-Stuart oscillators can be represented as the famous Kuramoto model [3-5]. The Kuramoto model is known for the synchronization of N-coupled oscillators. In our model, which is a special case of the Kuramoto and Landau-Stuart model, we can demonstrate the synchronization of *a*_1_ and *b*. By solving Eq. with and , we approximate to split the equations into their amplitudes and phase[[2]](#footnote-3). With the fixed amplitude of the *a*_1_ mode, $\alpha_{stable}$, the dynamics of the relative phase between the two modes, can be simplified as:

Here, we assume the detuning of the *b* mode is zero for simplicity $\Delta_{b}=0$. Equation takes the form of the Kuramoto model, and the critical value for synchronization is $G_{c}=\Gamma/2$ which corresponds to the condition for comb generation for the strong coupling case. While the actual dynamics of the relative phase are more complex and require solving the full equation as predicted in the previous discussion, this Kuramoto-like equation suggests that comb generation is in a state of pre-synchronization, with the two modes synchronized and being oscillating at $\omega_{p}/2$ under a large amount of pump power, as illustrated in Supplementary Supplementary Figure 4B. We observe several stages for routing to synchronization: At low pump power levels, the *a* and *b* modes are coupled to each other, leading to typical normal mode splitting as shown in Supplementary Fig. 3B, that is, the two spectral peaks are mainly separate by G. With a moderate pump power, they enter a state of quasiperiodic oscillations, generating combs (See Supplementary Supplementary Figure 3C-G). Eventually, they reach a state of frequency lock, achieving synchronization [6, 7]. In the pre-synchronized state, the potential do not have local minima in phases, thereby creating multiple beat notes which enable generation of combs in our case. We would like to emphasize that comb generations in our systems are fundamentally different from combs in other RF systems. Particularly, the works in Refs. [8, 9] employ three-wave miximg for comb generation while our combs are generated by harnessing the quasiperiodic motion, i.e., pre-synchronization of the two modes as discussed here.


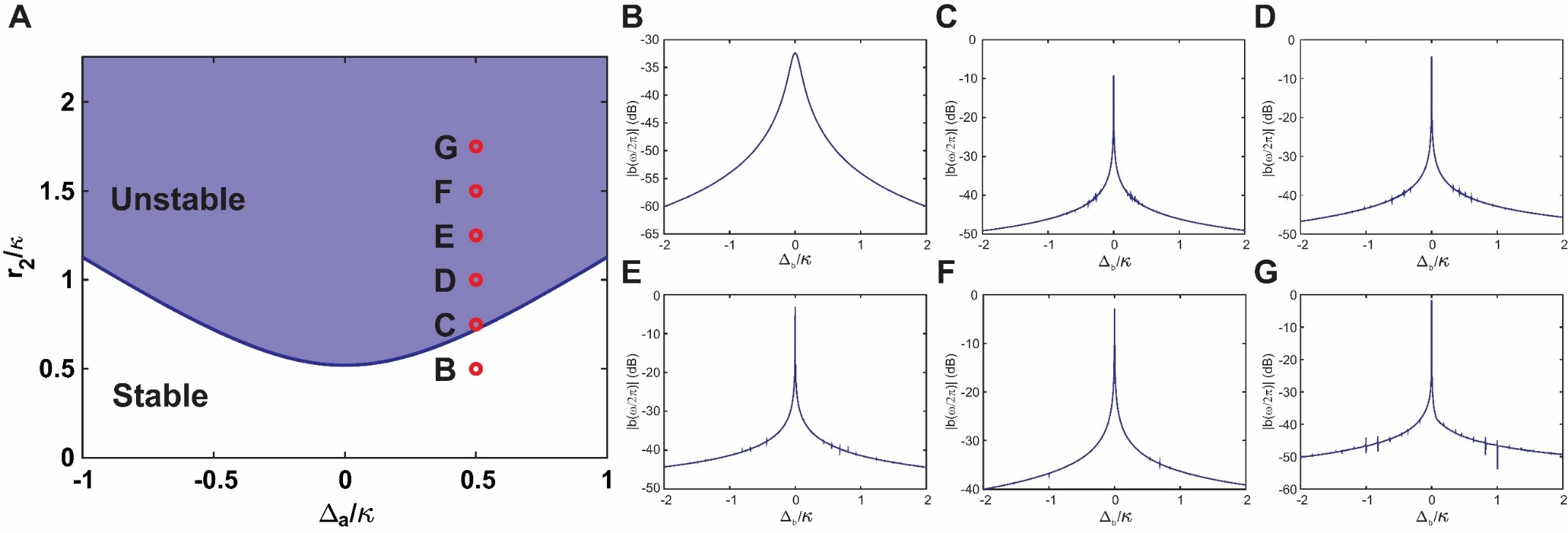


Supplementary Figure 2 A Phase diagram in $r_{2}$-$\Delta_{a}$ space for the weak coupling case $\left(G\ll\Gamma/2\right)$. The solid blue line indicates the relation of $r_{2}$_.crit_ and $\Delta_{a}$. The system is stable below the line (white area) while it becomes unstable above the line (blue shaded area) as the real part of the pole is larger than zero$\mathrm{Re}\left[s\right]>0$. B-G Spectrum of the mechanical mode $\left|b\left(\omega/2\pi\right)\right|$at the different $r_{2}$ values shown as red dots in panel A. No comb-spectra are observed, even after the trivial solution of the system becomes unstable. Note that the center frequency of the spectrum is $\omega_{a}$ in panel B, while the center frequency of the rest of the spectra in panels C-G is $\omega_{b}$ as predicted by Eq. and Eq. .


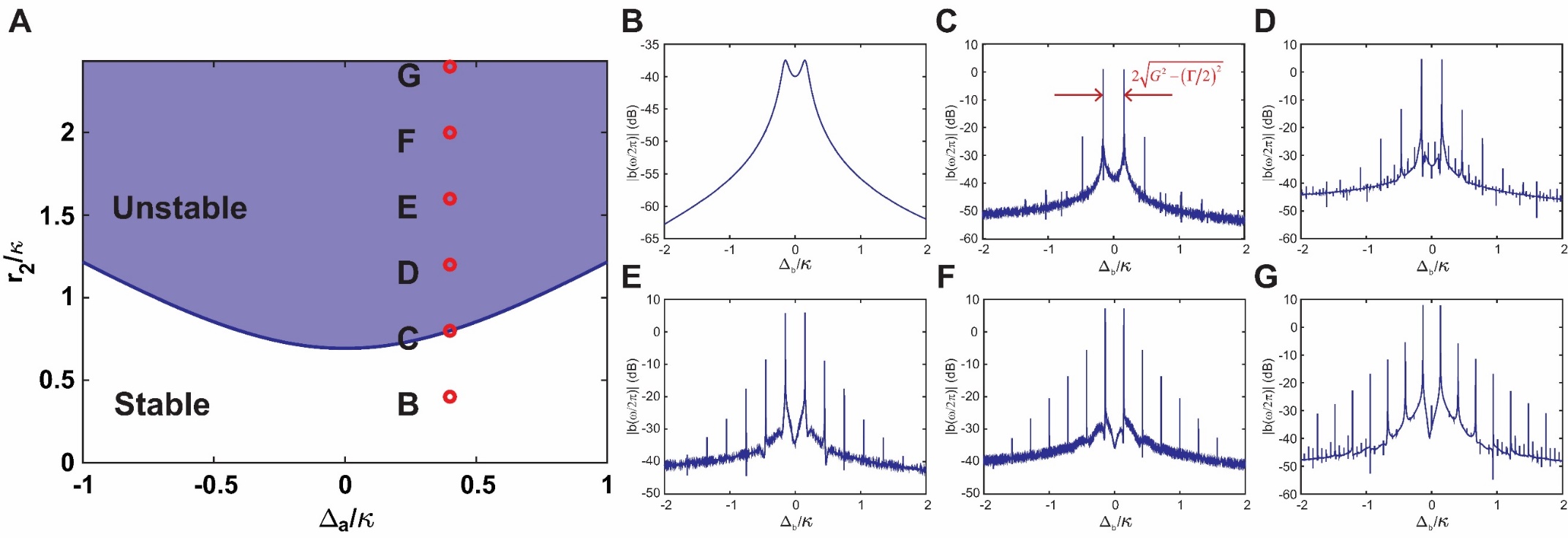


Supplementary Figure 3 A Phase diagram in $r_{2}$-$\Delta_{a}$ space for the strong coupling case $\left(G>\Gamma/2\right)$. The solid blue line indicates the relation of $r_{2}$_.crit_ and $\Delta_{a}$, The system is stable below the line (white area) while it becomes unstable above the line (blue shaded area) as the real part of the pole is larger than zero$\mathrm{Re}\left[s\right]>0$. B-G Spectrum of the mechanical mode $\left|b\left(\omega/2\pi\right)\right|$for the different $r_{2}$ values shown as red dots in panel A. The comb spectrum appears after the critical value of $r_{2}$, as shown in panel C.


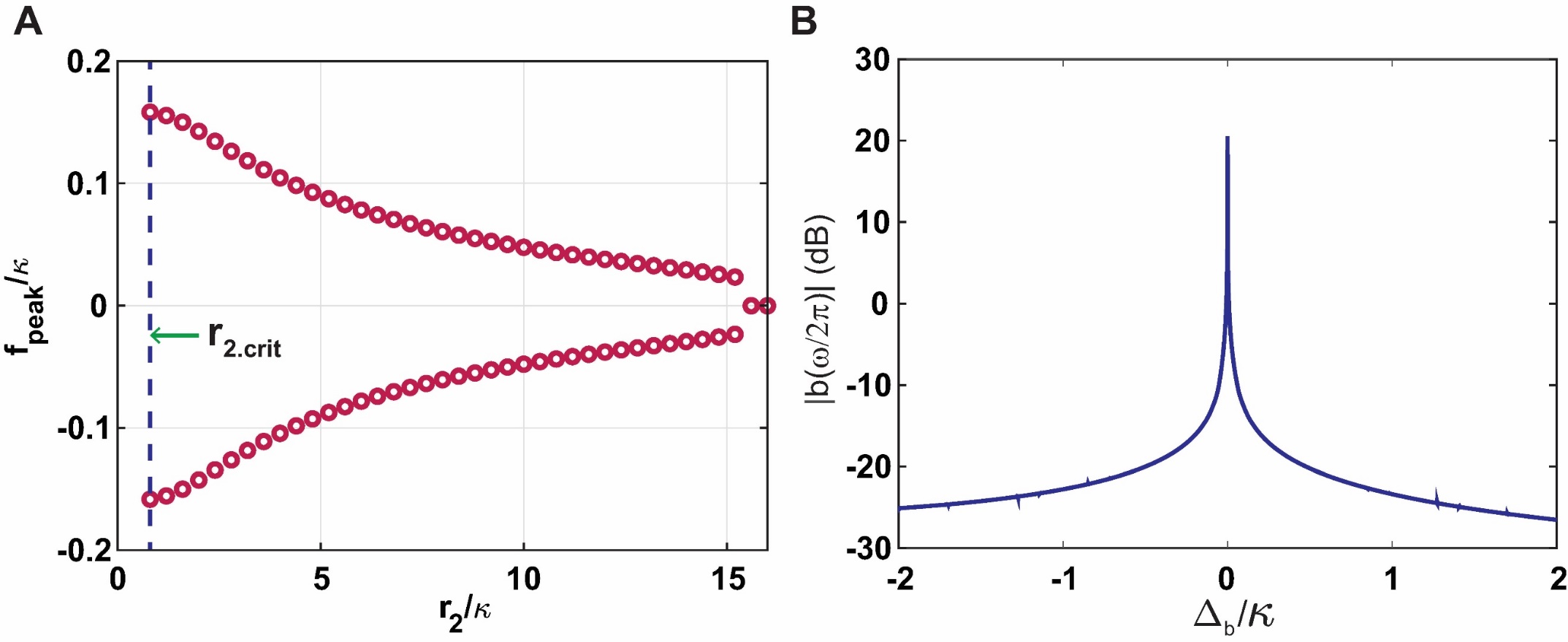


Supplementary Figure 4 A Representing frequency of two adjacent peaks in comb spectra with the small-signal gain parameter $r_{2}$. Combs appear after the critical value $r_{2}$_.crit_. The comb spacing decreases with $r_{2}$, and combs disappear after $r_{2}/\kappa=15.5$. B Spectrum of the mechanical mode at $r_{2}/\kappa=15.6$.

# Design, experimental setup and measurements

## qHT’s design

With regards to the design of the qHT, we followed the design procedure for parametric frequency dividers discussed in [10]. The main design goal is to achieve the lowest possible $P_{th}$. In order to do so, the following four resonant conditions must be satisfied, as in Supplementary Fig. 5:

1. The entirety of the mesh that includes the parallel connection of $L_{1}$ and $C_{1}$connected with series connection of $L_{3}$ and $C_{3}$, should series-resonate at $f_{p}$.
2. The parallel connection of $L_{2}$ and $C_{2}$, which is parallel resonant at $f_{p}$, must act as a notch-filter for the input signal at $f_{p}$.
3. For the signal generated around $f_{p}/2$ to be entirely delivered to the output port, the mesh that comprises the parallel connection of $L_{2}$ and $C_{2}$ connected with the series connection of $L_{3}$ and $C_{3}$, must series-resonate at $f_{p}/2$.
4. The parallel connection of $L_{1}$ and $C_{1}$, which is parallel resonant at $f_{p}/2$, must act as a notch-filter for the output signal at $f_{p}/2$.

We performed an optimization routine through a commercial Harmonic Balance [11] simulator as proposed in [10] to satisfy such listed four resonant conditions, thus obtaining the optimal value of the lumped components in the circuits ensuring the activation of the parametric instability with the lowest possible input power level. The commercial SAW resonator that we used in our qHT has a resonant frequency around $418 \text{MHz}$. Thus, the qHT is designed to be interrogated around twice the resonance frequency of the SAW device, thus around $836 \text{MHz}$. After designing and optimizing the circuit around these frequencies, the optimal values of inductors $L_{1}, L_{2}, L_{3}$ and $L_{out}$are found to be equal to $61 \text{nH}, 63 \text{nH}, 36 \text{nH}$ and $3.6 \text{nH}$, respectively. Meanwhile, the optimal values for the capacitance of the capacitors $C_{1}, C_{2}, C_{3}$ and $C_{out}$are found to be equal to $2.3 \text{pF}, 0.6 \text{pF}, 2.3 \text{pF}$ and $103 \text{pF}$, respectively. The measured admittance of the adopted SAW resonator is reported in Supplementary Fig. 6, where the resonant frequency is around $418 \text{MHz}$, the quality factor $Q\approx10,900$, the static capacitance $C_{0}\approx2.35 \text{pF}$and the electromechanical coupling coefficient $k_{t}^{2}\approx0.094\%$.

To verify the operation of our qHT, we built the circuit on a printed circuit board (PCB) using off-the-shelf lumped components. We run electromagnetic (EM) simulations for the entire PCB to take in account the parasitics from the board and from the SMA connectors at both the input and output ports. After executing the EM simulation, we adjusted the component values of the qHT to meet the optimal $P_{th}$ value while also ensuring compatibility with the commercially available discrete component values. The selected component values and their commercial part numbers are listed in Supplementary Table 2.


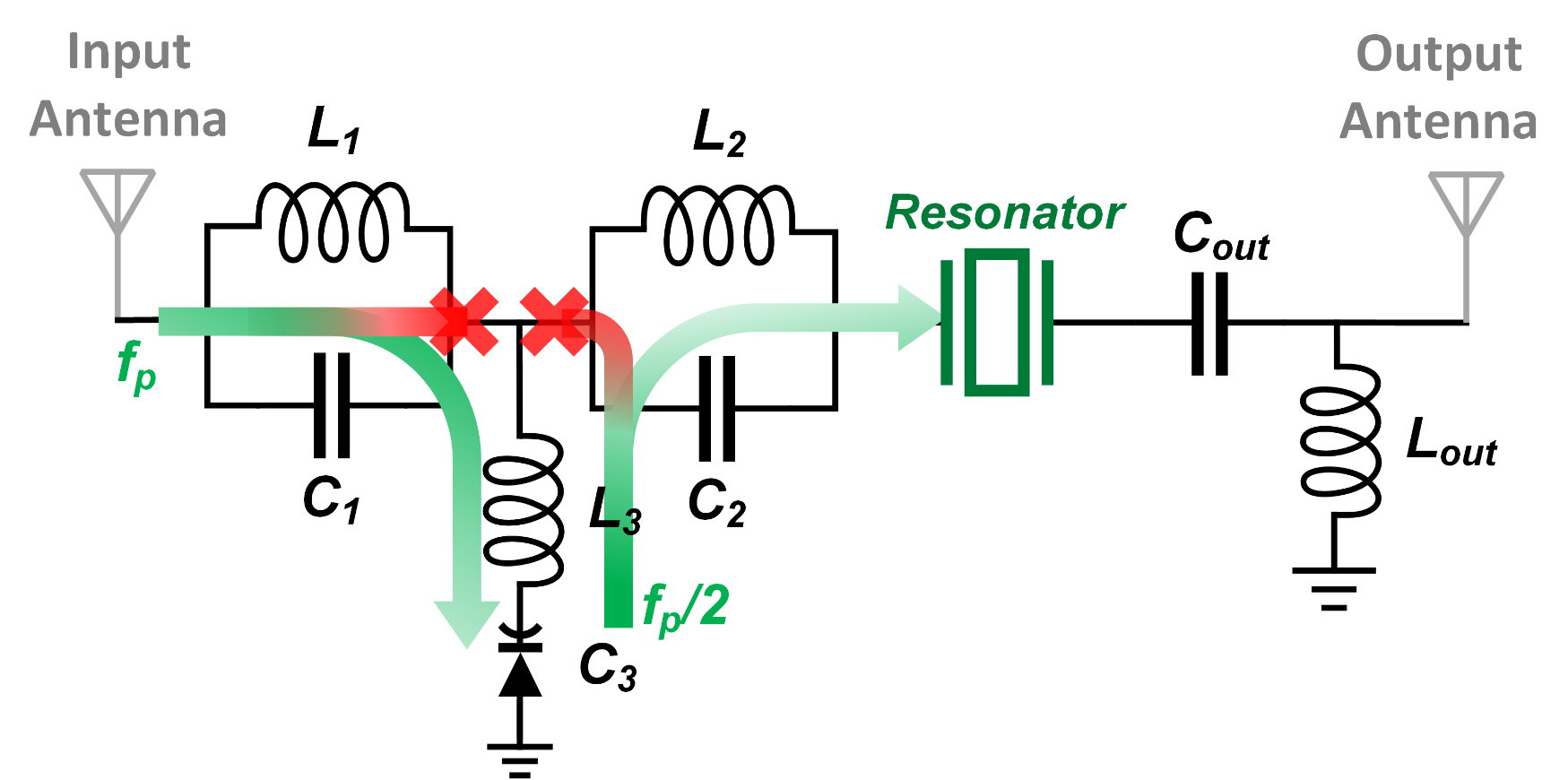


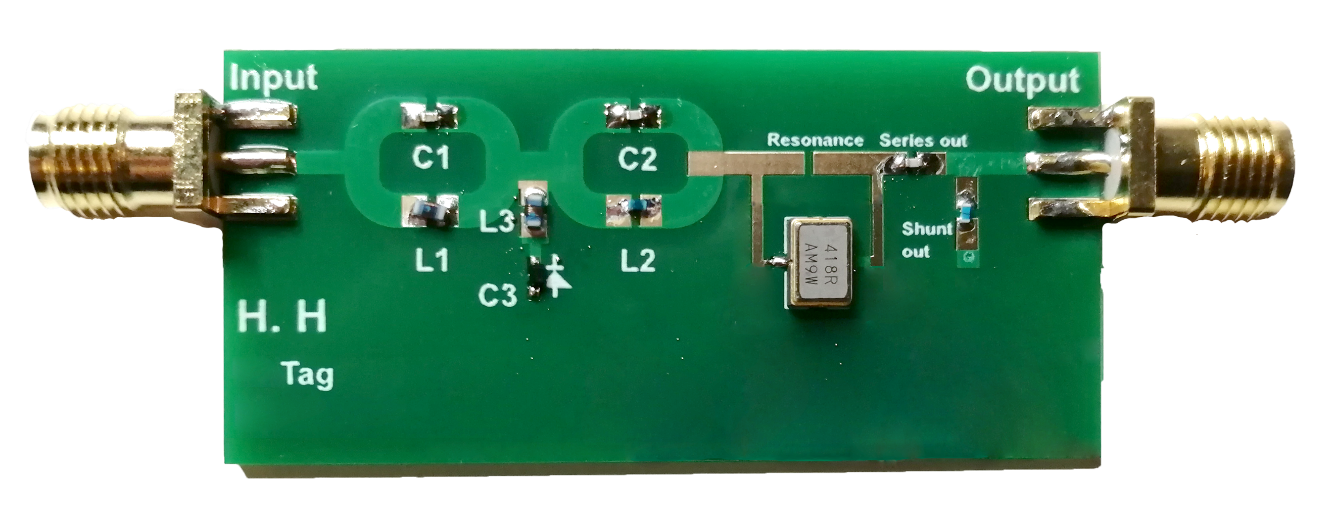


Supplementary Figure 5 Schematic of the built qHT showing the resonant conditions that must be satisfied to obtain the lowest possible $P_{th}$ value. A detailed illustrative diagram that represents the four essential prerequisites for the parametric circuit operation. A top-view picture of the built qHT’s board is also included.


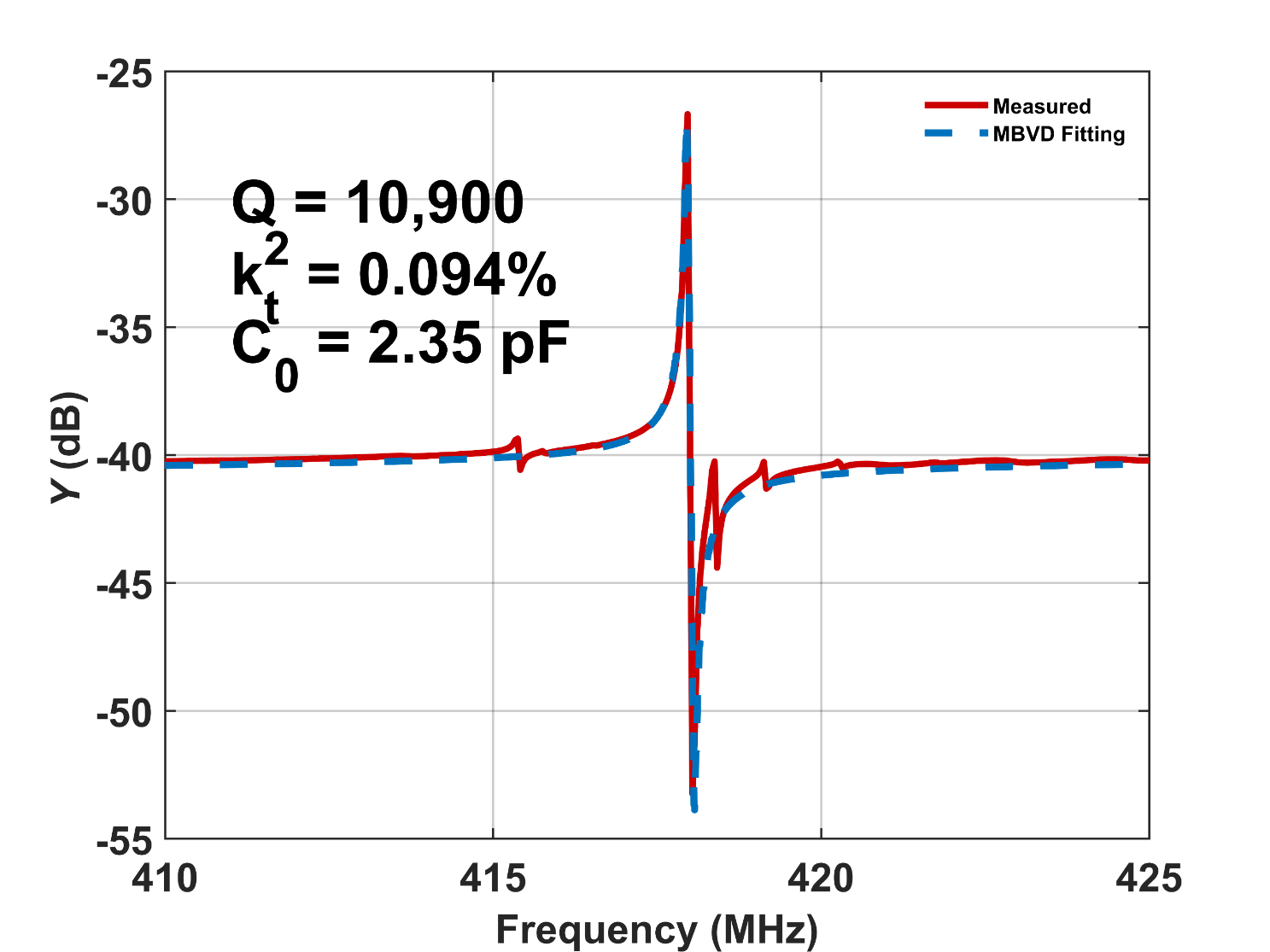


Supplementary Figure 6 The measured admittance of the SAW resonator adopted in this work vs. its extracted modified Butterworth-Van Dyke (MBVD) [12] fitting model.

Supplementary Table 2: Components list of the fabricated qHT:

| **Component** | **Nominal Value** | **Model Number** |
| --- | --- | --- |
| $L_{1}$ | 51 nH | 0603HP-51N |
| $L_{2}$ | 15 nH | 0603HP-15N |
| $L_{3}$ | 16 nH | 0603HP-16N |
| $L_{out}$ | 1 nH | 0402DC-1N0 |
| $C_{1}$ | 1.5 pF | GJM1555C1H1R5WB01 |
| $C_{2}$ | 1.8 pF | GJM1555C1H1R8WB01 |
| $C_{3}$ | 2.6 pF | SMV1405 |
| $C_{out}$ | 12 pF | GJM1555C1H120FB01 |
| SAW Resonator | 418 MHz | ASR418S2 |

## Wired experimental measurements

The experimental setup to characterize the performance of our qHT prototype is shown in Supplementary Fig. 7. We fed the input port of the qHT with a continuous-wave (CW) signal generated from a signal generator delivering a signal at 836.1 MHz, while we connected the output of the qHT to a spectrum analyzer to monitor the corresponding generated frequency comb. We connected a laptop to the signal generator to control the input power ($P_{r}$) received by the qHT and to perform the required power sweep at the input port automatically. Concurrently, the laptop was connected to the spectrum analyzer to store the generated frequency comb lines at every single swept $P_{r}$ value. The measured frequency combs at different $P_{r}$ levels are reported in Supplementary Fig. 8A, while the measured trend of $\Delta f$ vs. $P_{r}$ is reported in Supplementary Fig. 8B-C.

It is worth noting that the capacitance of the varactor employed in the proposed qHT has a nonlinear dependence on the applied modulation voltage as follows:

$$C\left( v \right)=C_{0}+C_{d1}V+C_{d2}V^{2}+\ldots$$

In this regard, we verified that the voltage magnitude across the varactor starts exceeding the varactor’s built-in voltage for $P_{r}\sim-8.5$dBm. As a result, for $P_{r}>-8.5$ dBm the varactor’s capacitance exhibits a strong third-order nonlinearity that inevitably causes higher ohmic losses in the qHT’s input mesh. Therefore, for our analytical model to be able to capture the trends of $\Delta f$ for $P_{r}$ both lower and higher than -8.5 dBm, we considered an additional power loss in our model that captures the increase in ohmic dissipations affecting the varactor for $P_{r}>-8.5$ dBm, thus when its voltage approaches and exceeds the built-in voltage [13].


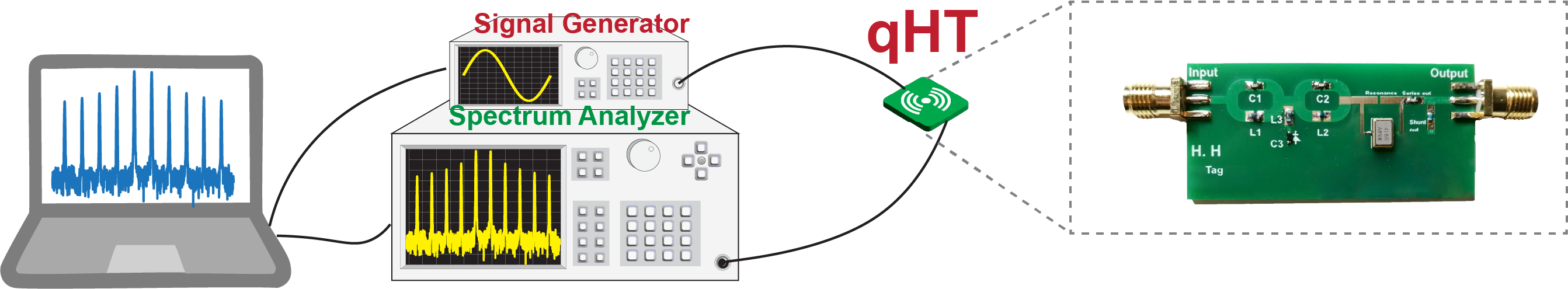


Supplementary Figure 7 A schematic of the wired experimental setup. A signal generator connected directly to the input of the qHT and a spectrum analyzer connected to the output of the qHT. A laptop is connected to the signal generator to sweep the input power level, while simultaneously monitoring and storing the generated frequency comb from a spectrum analyzer.


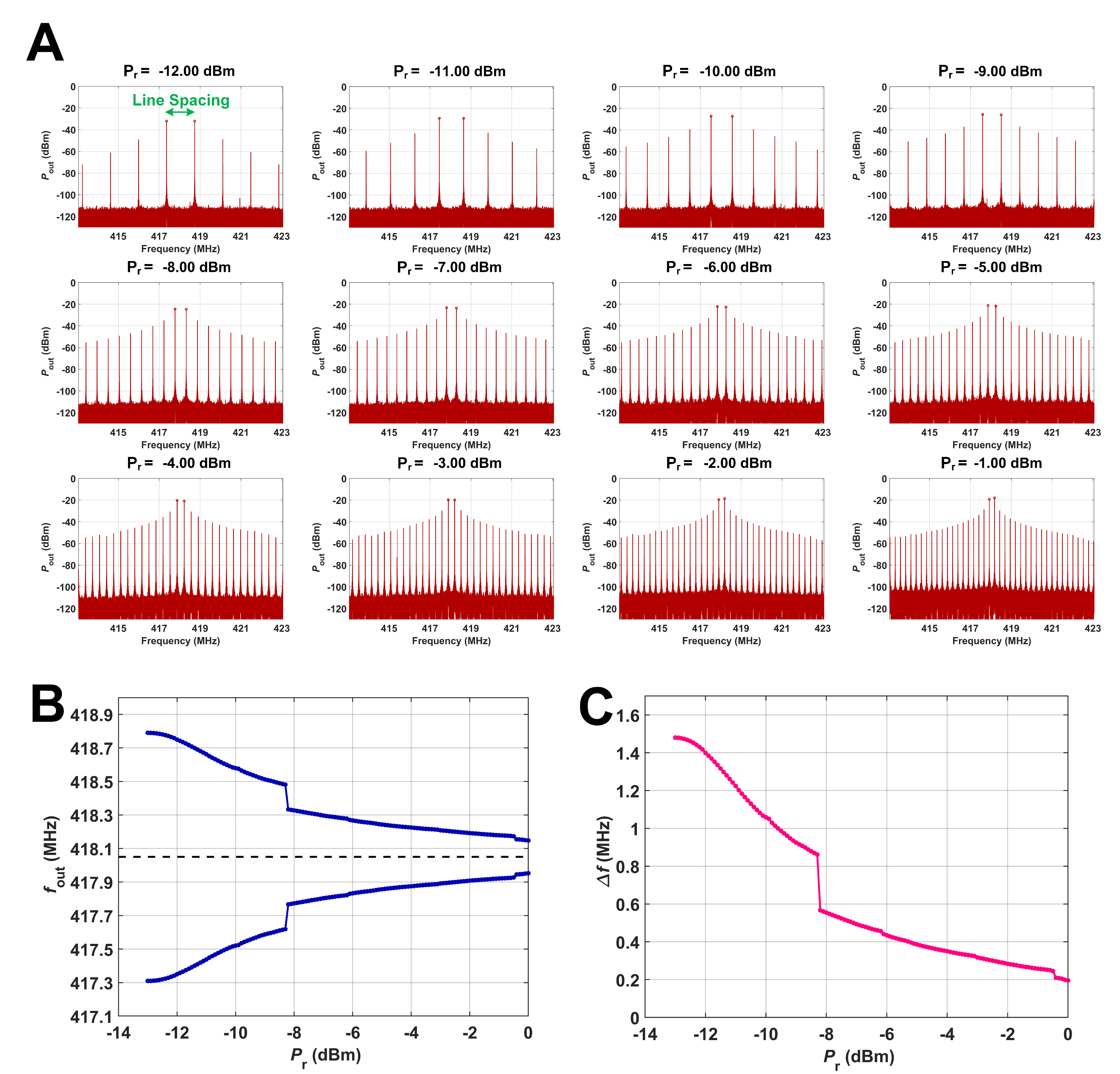


Supplementary Figure 8 A Measured frequency combs generated from the qHT during the wired characterization at different $P_{r}$ values. B The frequency of the two strongest frequency comb lines is plotted vs. $P_{r}$. C $\Delta f$ is plotted vs. $P_{r}$.

## Wireless experimental measurements with the drone

We conducted the wireless characterization of the qHT when mounted on a drone by performing a wireless testing as shown in Supplementary Fig. 10. Also, the real-time performed experiment is shown in Supplementary Video 1. The main purpose of this experiment was to emulate a transceiver representing an interrogator/reader point, sending an interrogating signal to excite the qHT and reading out the resulting frequency comb. To achieve this, we transmitted the continuous wave (CW) interrogating signal through a directive antenna (Yagi-Uda Antenna) after amplifying it with a power amplifier. Additionally, we received the backscattered frequency comb signal through an antenna connected to a spectrum analyzer. The spectrum analyzer sent all the measured frequency comb data to a laptop for the automatic extraction of the comb-line spacing.

We mounted the qHT on-board of a drone flying at the same height as the interrogating/reading antennas, as shown in Supplementary Fig. 10. Then we placed a meter tape between the qHT and the beacon to physically measure the distance (d) for any considered distance value. The TX CW signal was transmitted at 836.1 MHz, and the effective isotropic radiated power (EIRP) level was kept fixed at around +38 dBm. Then, we remotely controlled the drone and made it to fly, moving it with a fixed step of 10 cm while recording the corresponding frequency comb for each distance together with the actual drone’s distance according to the ruler. We constructed a graph showing the variation in $\Delta f$ versus the measured distance, as demonstrated in Supplementary Fig. 11.

To obtain a more accurate assessment of the performance of the qHT, we conducted an additional “stationary-drone” ranging experiment (See Fig.4 d-e in the main manuscript), as illustrated in Supplementary Fig. 12. We used the same interrogation node set-up as in Supplementary Fig. 10, but we manually relocated the drone to different positions away from the interrogation node and along the TX antenna’s direction, Meanwhile, we recorded the frequency comb spectrum at each position, as well as the drone’s distance according to the ruler. By repeating the same experimental procedure as in Supplementary Fig. 10, we obtained a new trend of $\Delta f$ versus distance, as shown in Supplementary Fig. 12. Notably, the ranging error in the “stationary-drone” experiment was significantly lower than that of the flying drone experiment, as evident in Supplementary Fig. 12D.


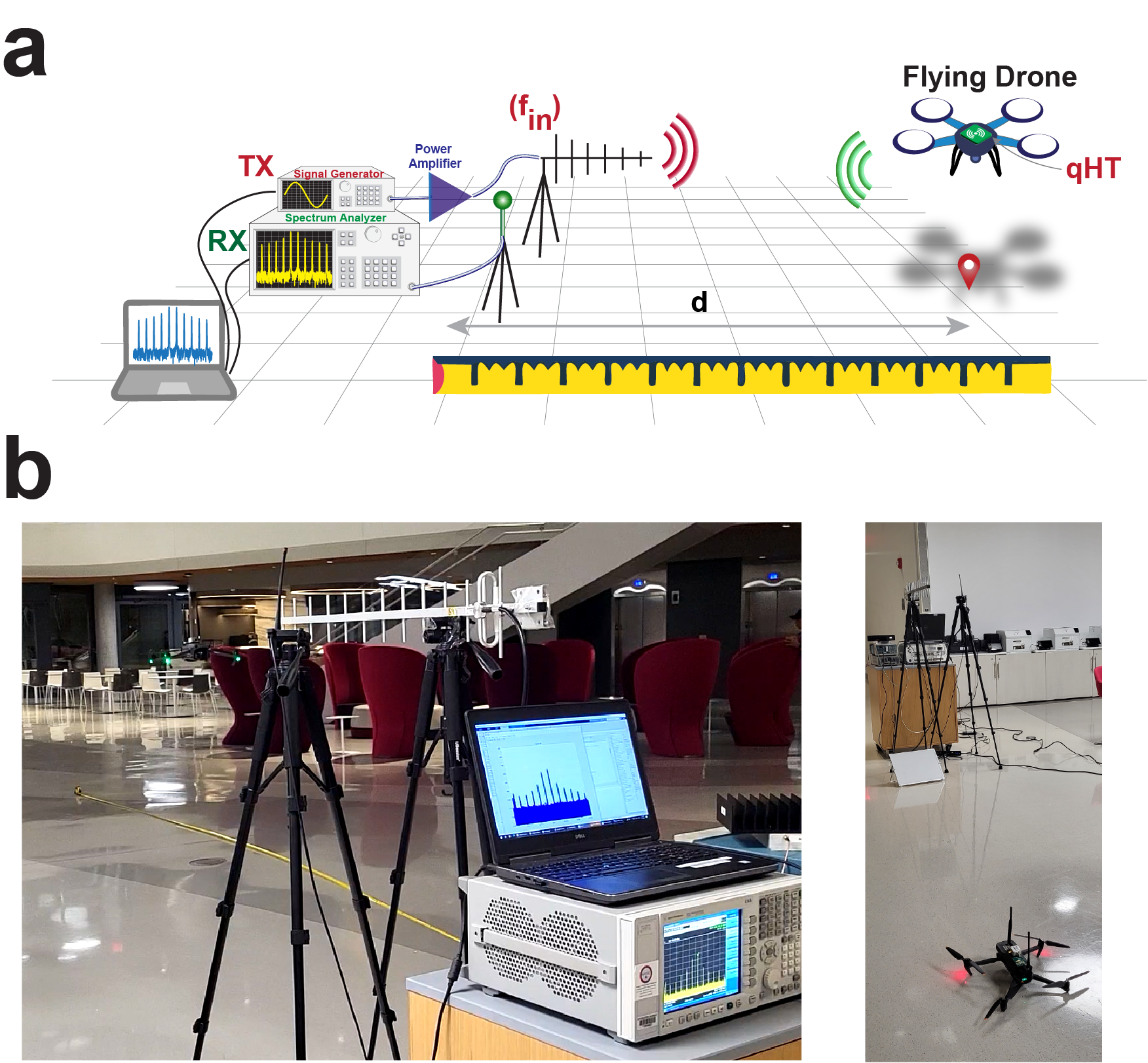


Supplementary Figure 9 A Schematic of the experimental setup used for the drone ranging experiment. A signal generator connected to a power amplifier and to a directive antenna represents the TX that interrogates the qHT. The backscattered signal is captured via an antenna through a spectrum analyzer and recorded through a laptop for every single distance. B Photos of the real experimental setup that took place in a hall of the Interdisciplinary Science and Engineering Complex (ISEC) at Northeastern University.

| **A**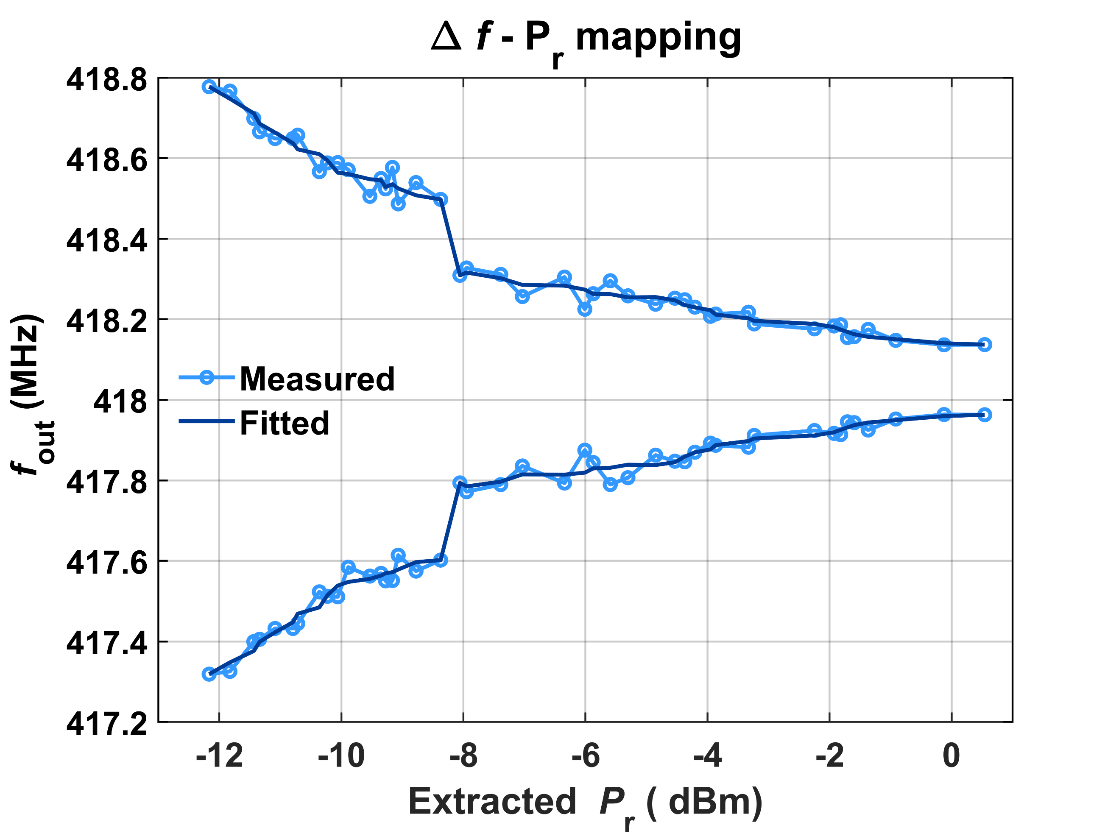 | **B**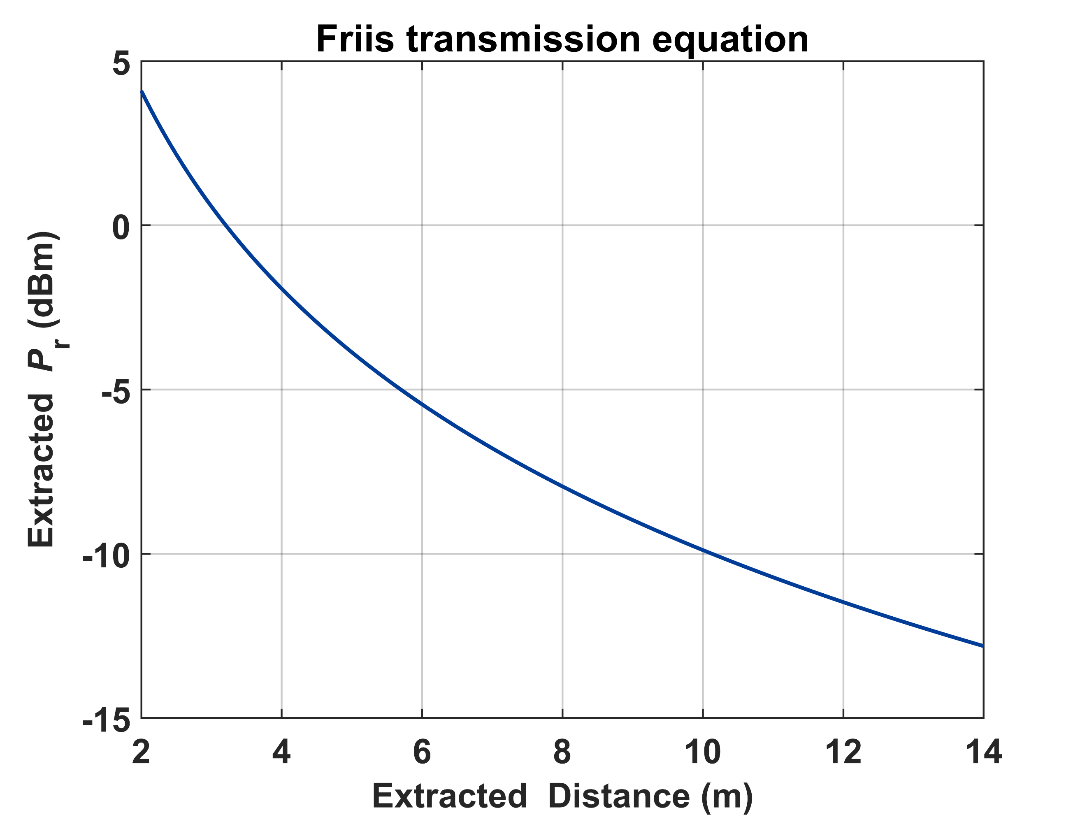 |
| --- | --- |
| **C 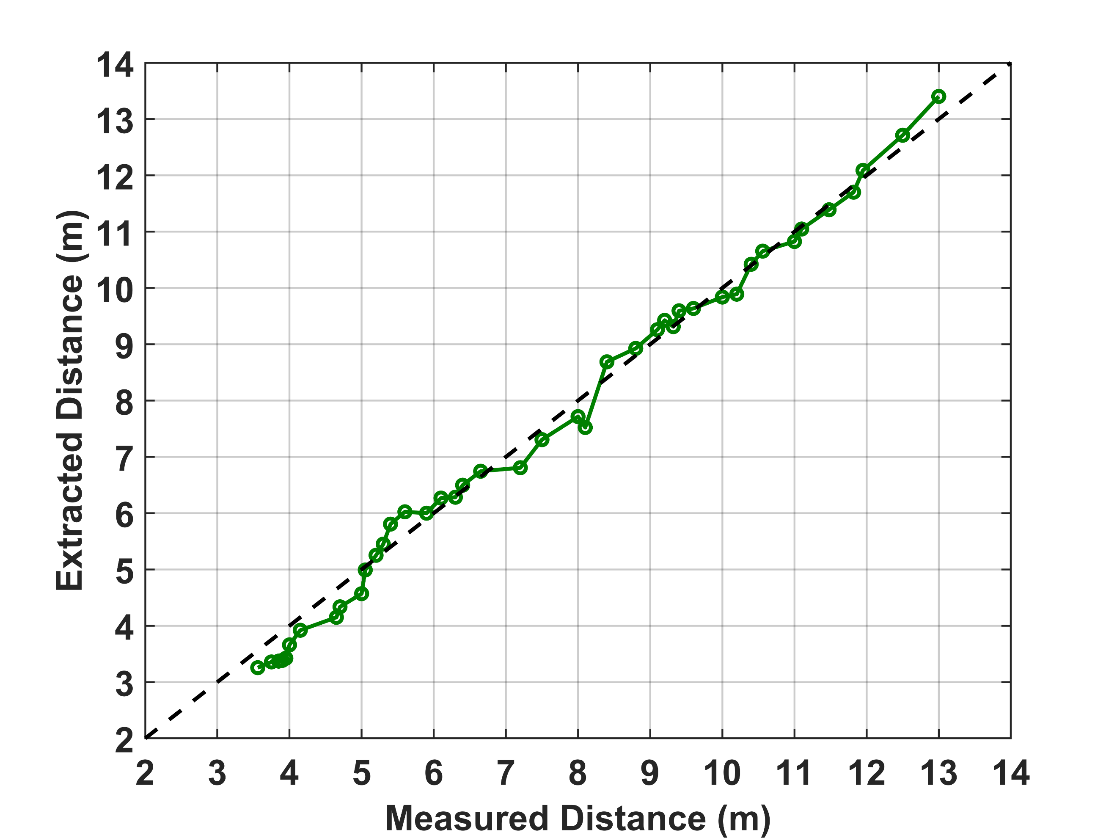** | **D 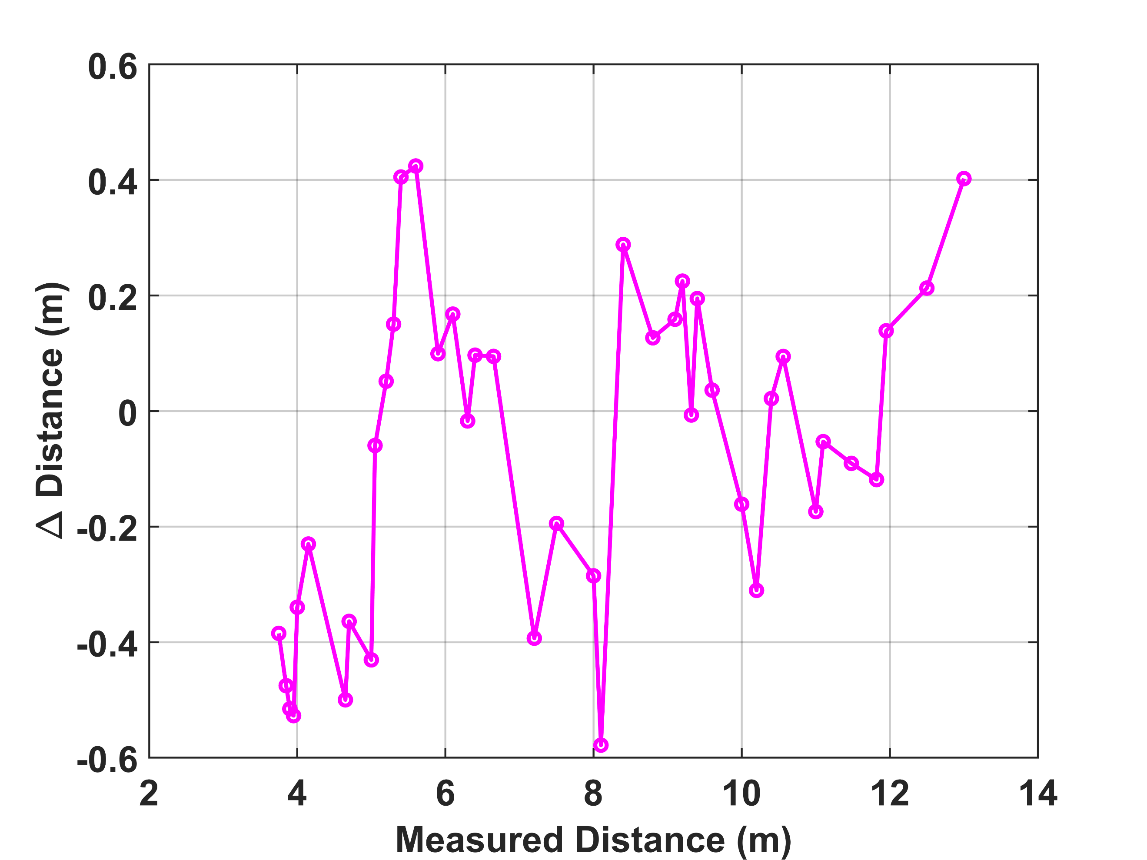** |

Supplementary Figure 10 A Measured frequency for the two strongest comb lines in the flying drone experiment vs. the corresponding extracted $P_{r}$ values. B The Friis transmission curve of the extracted $P_{r}$values vs. the corresponding extracted distance. C Extracted drone’s distance using the Friis transmission in B vs. the ruler-measured distance. D Error in the extracted drone’s distance compared to the drone’s ruler-measured distance.


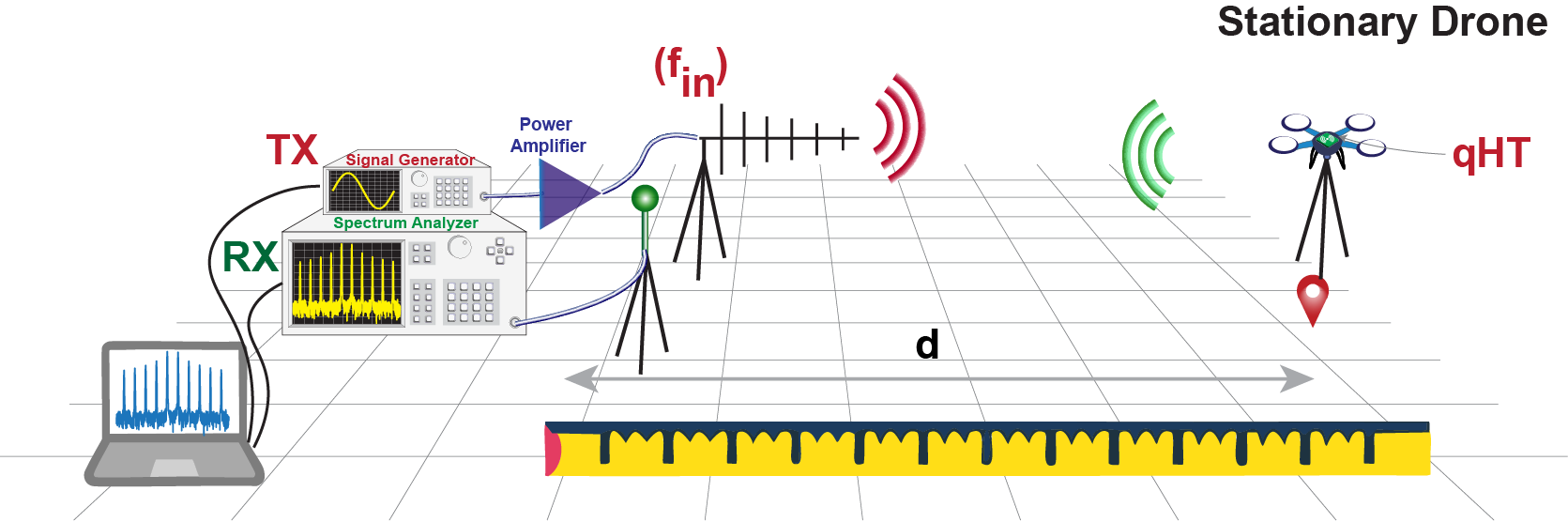


Supplementary Figure 11 A detailed schematic of the experimental setup for the “stationary-drone” ranging experiment. A signal generator connected to a power amplifier connected to a directive antenna are forming the TX that interrogates the qHT. The backscattered signal is captured via an antenna through a spectrum analyzer and recorded by using a laptop for every single distance.

| **A**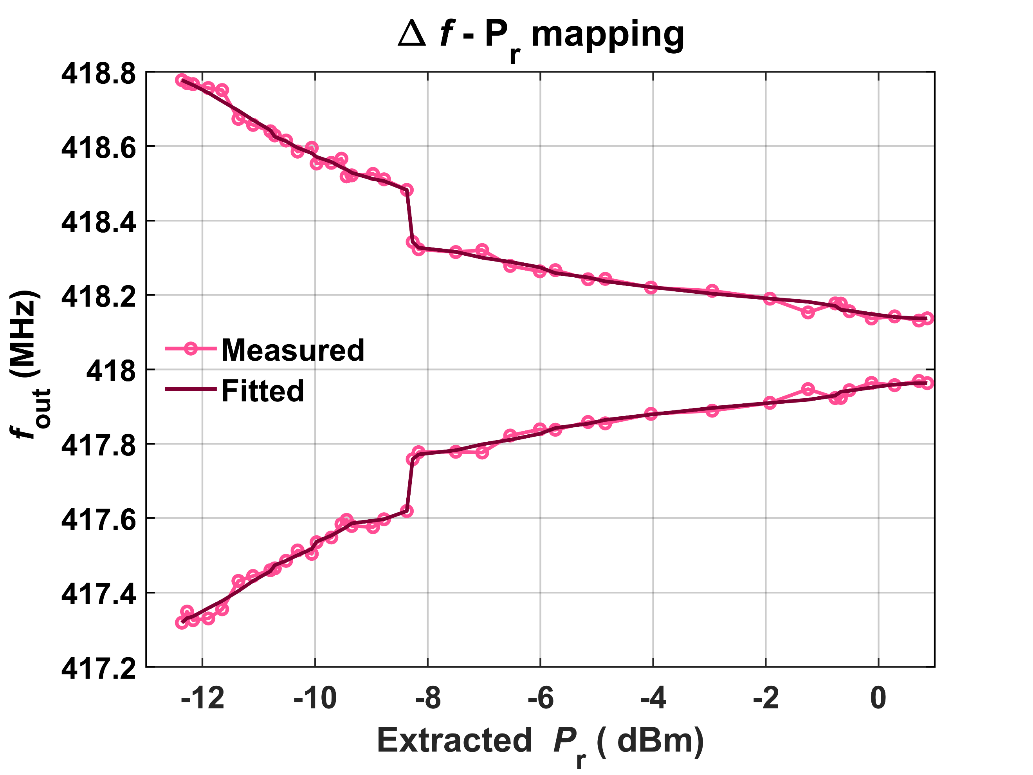 | **B**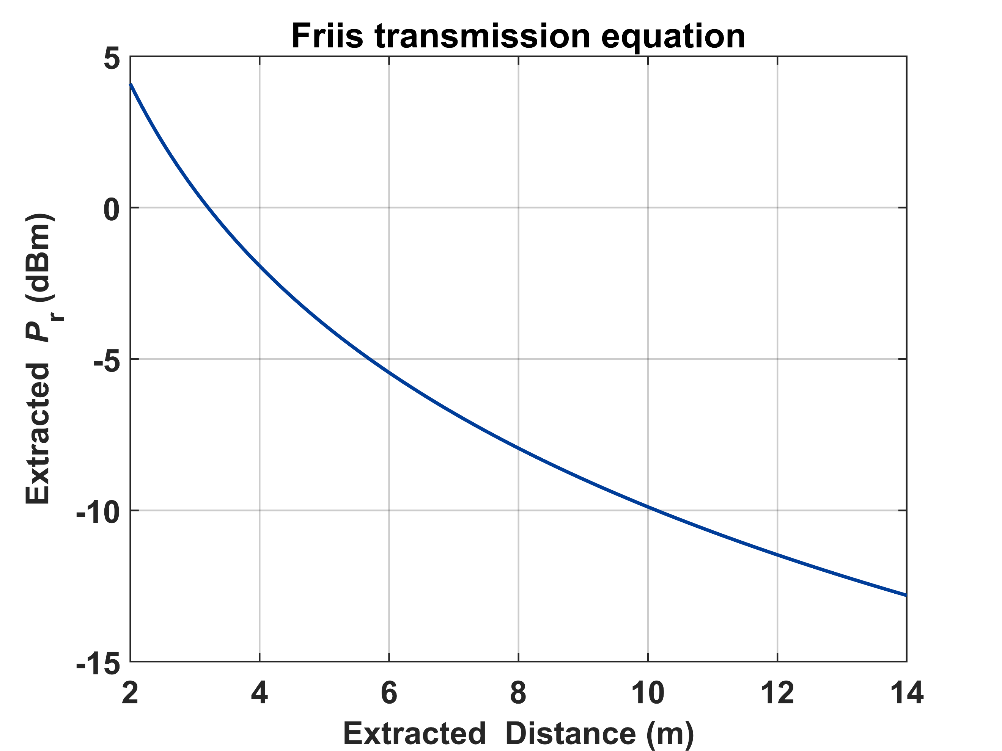 |
| --- | --- |
| **C** 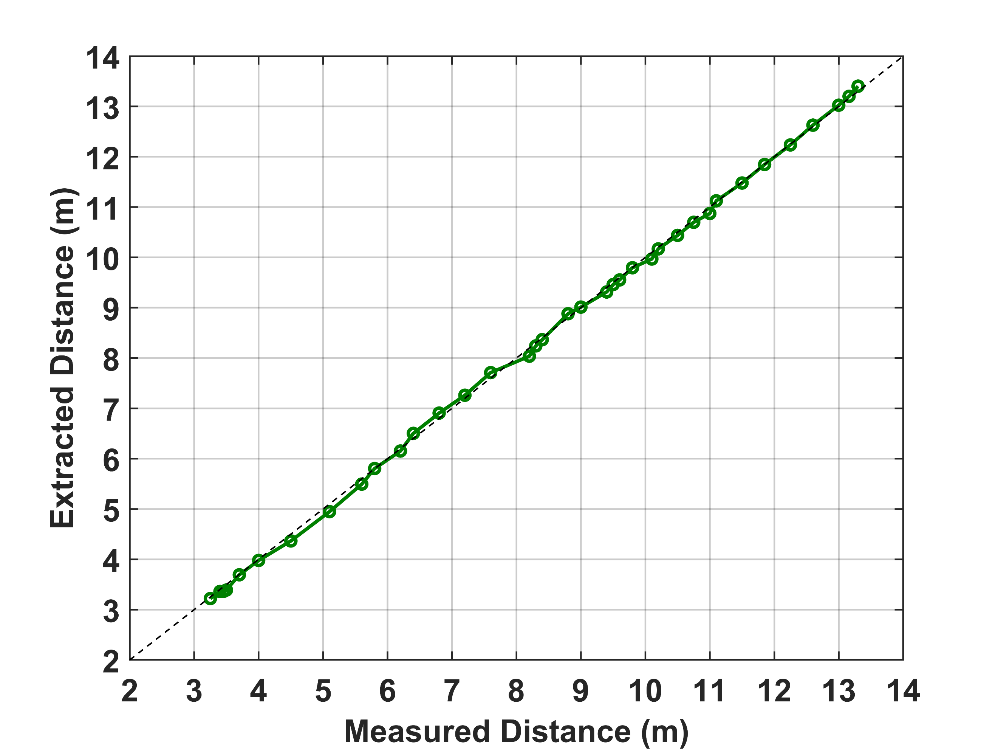 | **D**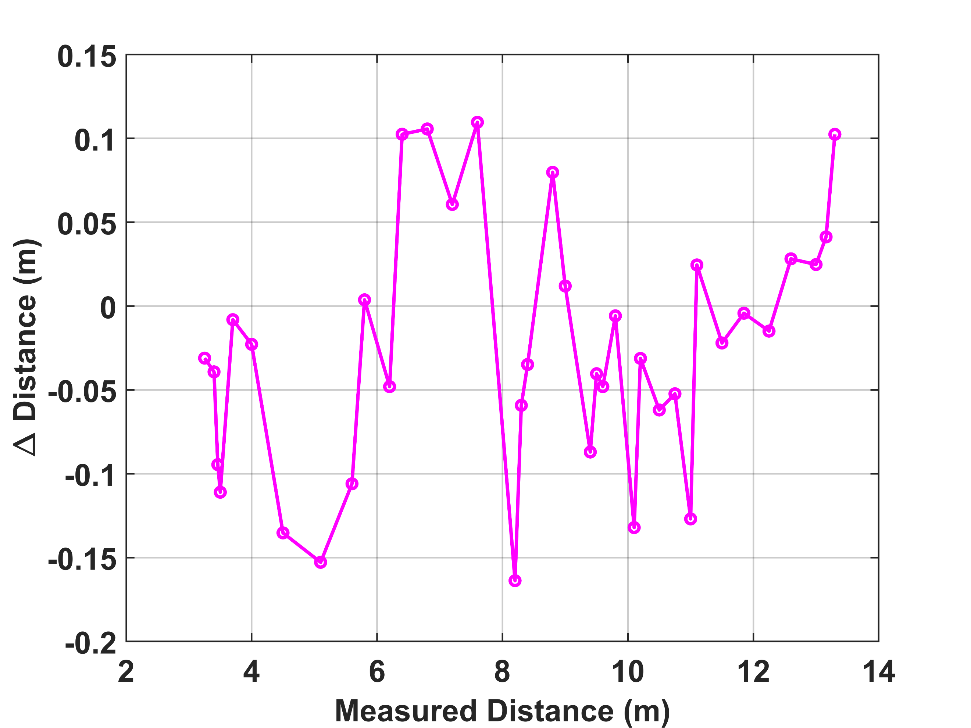 |

Supplementary Figure 12 A Measured frequency of the two strongest comb lines generated from the qHT during the “stationary-drone” experiment vs. the corresponding extracted $P_{r}$values. B The Friis transmission curve of the extracted $P_{r}$values vs. the corresponding extracted distance. C Drone’s distance extracted from the Friis transmission equation shown in Supplementary Fig. 10B vs. the drone’s ruler-measured distance. D Error in the extracted drone’s distance compared to the drone’s ruler-measured distance.

## Performance comparison with the state-of-the-art

A comparison of the ranging performance of the qHT reported in this work with those of other reported wireless sensing systems is provided in Supplementary Table 3. While the authors in [14] reported an accuracy of 0.58 cm, it's important to note that they had to capture and average their measured signal 400 times to achieve this level of accuracy. This renders the system unsuitable for real-time applications, as it introduces overhead in terms of power consumption and signal processing. Also, no averaging was implemented in our experimental characterization. Moreover, in order to optimize the ranging accuracy, [14] required using six different interrogation frequencies distributed across a wide bandwidth. Similarly, Refs. [15, 16] relied on several tags (up to 25). Nonetheless, the accuracy achieved in Refs. [15, 16] was limited to 21 cm with a read-range of only 3 meters. The passive tags in [17] were able to achieve an accuracy of 5 cm with a limited range of 2.7 meters. However, this was possible only when operating in an anechoic chamber.

Supplementary Table 3: Comparison with other related works for Far-Field Ranging

| Ref | Tag Type | Technology | Range  (m) | Accuracy | Relative Error =  Max Error / Max Range | No. of Interrogation Frequencies | Testing Environment | EIRP (dBm) | No. of employed tags |
| --- | --- | --- | --- | --- | --- | --- | --- | --- | --- |
| **This work** | **Passive without IC chip** | **Frequency Comb** | **13** | **< 16 cm**  **RMS = 7.7 cm** | **0.16/13 = 0.0123** | **1** | **Indoor** | **38** | **1** |
| [14] | Passive without IC | Harmonic Tag | 1.1 | < 0.58 cm** | 0.058/1.1 = 0.0527 | 4 to 6 | Indoor | 36 | 1 |
| [18] | Passive RFID with IC chip | UHF RFID | 6.5 | < 50 cm | 0.2/6.5 = 0.031 | 1 | Indoor | - | 200 |
| [15] | Passive RFID with IC chip | UHF RFID | 0.8 | <8.5 cm | 0.085/0.8 = 0.106 | 1 | Indoor | 39 | 3 |
| [16] | Passive RFID with IC chip | UHF RFID | 3 | < 21 cm | 0.21/3= 0.07 | 1 | Indoor | 35 | 25 |
| [19] | Passive RFID with IC chip | UHF RFID | n/a | < 50 cm | n/a | 1 | Indoor | 39 | 1 |
| [20] | Passive RFID with IC chip | UHF RFID | 4.7 | < 6.8 cm | 0.068/4.7 = 0.0144 | 13 | Anechoic Chamber | - | 1 |
| [17] | Passive RFID with IC chip | UHF RFID | 2.7 | < 5 cm | 0.05/2.7 = 0.0185 | >1 | Anechoic Chamber | - | 1 |
| [21] | Passive | Harmonic Tag | 4 | < 40 cm | 0.4/4 = 0.1 | 2 | Simulation | 30 | 1 |
| [22] | Active Motes with IC chips | Wireless Sensor Motes | 9.5 | < 3.4 m | 3.4/9.5 = 0.35 | n/a | Indoor | - | 1 |

**Note that the authors of this paper captured and averaged the signal 400 times by oscilloscope.

## Relation between the electromechanical coupling G and *k*_t_^2^

In the main text, we discussed the relation of G and *k*_t_^2^, and we thoroughly derive it as following. The internal energy of piezoelectric systems may be written by:

$$U =\frac{1}{2}\sum S_{ij}^{E}X_{i}X_{j}+\sum d_{mi}E_{m}X_{i}+\frac{1}{2}\sum\varepsilon_{mk}^{X}X_{k}X_{m}.$$

The first term in Eq. indicates the mechanical energy $U_{MM}$, while the third term represents the electrical stored energy $U_{EE}$. Interestingly, the second term shows the interaction between the mechanical and electrical modes via piezoelectricity, and we denote as $U_{ME}$ the energy converted from electrical to mechanical domains, or vice versa. As a result, the electromechanical coupling coefficient is given by [23]:

$$k_{t}^{2}=\frac{U_{ME}}{\sqrt{U_{MM}U_{EE}}}.$$

We consider the equivalent piezoelectric systems in quantum mechanical language and present the Hamiltonian.

Here $a \left(or a^{\dagger}\right)$ and $b \left(or b^{\dagger}\right)$ are annihilation (or creation) operators for the modes *a* and *b*, which are quantum-mechanical counterparts to their amplitudes. The terms in Eq. represent the mechanical, electrical and conversion energies as discussed above. So we can naively plug them into Eq. .

$$k_{t}^{2}\approx G\sqrt{\frac{N_{a}N_{b}}{2\omega_{a}\omega_{b}N_{a}N_{b}}}$$

where $N_{a}=a^{\dagger}a$ and $N_{b}=b^{\dagger}b$ are the number operators of the electrical and mechanical modes, respectively. Assuming they have similar frequencies $\omega_{a}\approx\omega_{b}$, we get the simplified relationship $G\approx\sqrt{2}k_{t}^{2}\omega_{b}$which has been used in the main manuscript.

## Relation between the quality factor and the dynamic range

The use of the MEMS resonator is the key component for the proper operation of our circuit, serving as the fundamental element enabling the existence of frequency-comb states. As evident from the following figure depicting the response of a circuit simulation we run using time-domain methods, the MEMS resonator must possess a minimum Q value for the frequency combs to be generated. While this minimum Q value is around 60, a much higher Q is needed to really be able to use qHT for ranging measurements in actual operative scenarios. In fact, the MEMS resonator’s Q also dictates the dynamic range of qHTs, which directly translates into the difference between the maximum and the minimum distance that qHTs can cover for a fixed EIRP value used for the interrogation signal, as depicted on the right y-axis of the following figure. In other words, with a MEMS resonator having a Q of 70, we would have frequency-comb generation at only one power level. Obviously, this prevents from being really able to measure the distance of an item placed within a wide area around the interrogation device. It is also worth emphasizing that replacing the MEMS resonator with a lumped LC resonator would make it impossible to generate the same pre-synchronization states that are responsible for the generation of our frequency combs, regardless of the Q of the LC component. This is due to the relatively low inductance that can be synthesized by using the available inductor technologies, which has to be limited to few micro Henries (rather than the mHs granted by the MEMS resonator) to ensure that the pump frequency remains higher than the self-resonance frequency of the inductor when operating with a pump signal in the Ultra-High-Frequency range.


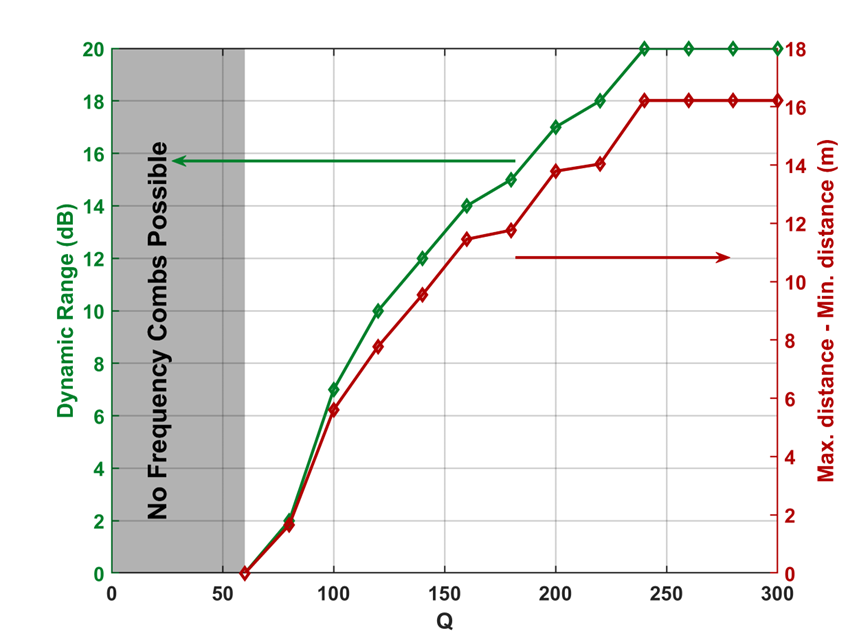


Supplementary Figure 13 A simulation result showing the minimum required Q value for the generation of the frequency comb. The plot also shows that in order to obtain a practical dynamic range, the Q value should be large enough to achieve that. The right y-axis shows the maximum – minimum achievable range for the same range of Q values.

# References

[1] A. Rodriguez, M. Soljacic, J. D. Joannopoulos, and S. G. Johnson, “chi((2)) and chi((3)) harmonic generation at a critical power in inhomogeneous doubly resonant cavities,” *Optics Express,* vol. 15, no. 12, pp. 7303-7318, Jun 11, 2007.

[2] S. Khan, and H. E. Tureci, “Frequency Combs in a Lumped-Element Josephson-Junction Circuit,” *Physical Review Letters,* vol. 120, no. 15, Apr 9, 2018.

[3] Y. Kuramoto, "Self-entrainment of a population of coupled non-linear oscillators." pp. 420-422.

[4] S. H. Strogatz, “From Kuramoto to Crawford: exploring the onset of synchronization in populations of coupled oscillators,” *Physica D,* vol. 143, no. 1-4, pp. 1-20, Sep 1, 2000.

[5] S. Gupta, A. Campa, and S. Ruffo, “Kuramoto model of synchronization: equilibrium and nonequilibrium aspects,” *Journal of Statistical Mechanics: Theory and Experiment,* vol. 2014, no. 8, 2014.

[6] A. Pikovsky, M. Rosenblum, and J. Kurths, *Synchronization : a universal concept in nonlinear sciences*, Cambridge: Cambridge University Press, 2001.

[7] S. H. Strogatz, *Nonlinear dynamics and chaos : with applications to physics, biology, chemistry, and engineering*, Second edition. ed., Boulder, CO: Westview Press, a member of the Perseus Books Group, 2015.

[8] S. Khan, and H. E. Türeci, “Frequency Combs in a Lumped-Element Josephson-Junction Circuit,” *Physical Review Letters,* vol. 120, no. 15, pp. 153601, 04/09/, 2018.

[9] P. Lu, T.-C. Chien, X. Cao, O. Lanes, C. Zhou, M. J. Hatridge, S. Khan, and H. E. Türeci, “Nearly Quantum-Limited Josephson-Junction Frequency-Comb Synthesizer,” *Physical Review Applied,* vol. 15, no. 4, pp. 044031, 04/20/, 2021.

[10] H. M. E. Hussein, M. A. A. Ibrahim, G. Michetti, M. Rinaldi, M. Onabajo, and C. Cassella, “Systematic Synthesis and Design of Ultralow Threshold 2:1 Parametric Frequency Dividers,” *Ieee Transactions on Microwave Theory and Techniques,* vol. 68, no. 8, pp. 3497-3509, Aug, 2020.

[11] A. Suarez, and R. Melville, “Simulation-assisted design and analysis of varactor-based frequency multipliers and dividers,” *Ieee Transactions on Microwave Theory and Techniques,* vol. 54, no. 3, pp. 1166-1179, Mar, 2006.

[12] H. Jin, S. R. Dong, J. K. Luo, and W. I. Milne, “Generalised Butterworth-Van Dyke equivalent circuit for thin-film bulk acoustic resonator,” *Electronics Letters,* vol. 47, no. 7, pp. 424-425, Mar 31, 2011.

[13] M. Cotrufo, S. A. Mann, H. Moussa, and A. Alu, “Nonlinearity-Induced Nonreciprocity-Part I,” *Ieee Transactions on Microwave Theory and Techniques,* vol. 69, no. 8, pp. 3569-3583, Aug, 2021.

[14] Y. F. Ma, and E. C. Kan, “Accurate Indoor Ranging by Broadband Harmonic Generation in Passive NLTL Backscatter Tags,” *Ieee Transactions on Microwave Theory and Techniques,* vol. 62, no. 5, pp. 1249-1261, May, 2014.

[15] J. Zhang, X. Wang, Z. Yu, Y. Lyu, S. Mao, S. C. G. Periaswamy, J. Patton, and X. Wang, “Robust RFID Based 6-DoF Localization for Unmanned Aerial Vehicles,” *IEEE Access,* vol. 7, pp. 77348-77361, 2019.

[16] S. Azzouzi, M. Cremer, U. Dettmar, R. Kronberger, and T. Knie, "New measurement results for the localization of UHF RFID transponders using an Angle of Arrival (AoA) approach." pp. 91-97.

[17] M. Scherhaufl, M. Pichler, D. Muller, A. Ziroff, and A. Stelzer, “Phase-of-Arrival- Based Localization of Passive UHF RFID Tags,” *2013 Ieee Mtt-S International Microwave Symposium Digest (Ims)*, 2013.

[18] J. Wang, and D. Katabi, “Dude, Where's My Card? RFID Positioning That Works with Multipath and Non-Line of Sight,” *Acm Sigcomm Computer Communication Review,* vol. 43, no. 4, pp. 51-62, Oct, 2013.

[19] J. Zhang, Y. B. Lyu, J. Patton, S. C. G. Periaswamy, and T. Roppel, “BFVP: A Probabilistic UHF RFID Tag Localization Algorithm Using Bayesian Filter and a Variable Power RFID Model,” *Ieee Transactions on Industrial Electronics,* vol. 65, no. 10, pp. 8250-8259, Oct, 2018.

[20] V. Viikari, P. Pursula, and K. Jaakkola, “Ranging of UHF RFID Tag Using Stepped Frequency Read-Out,” *Ieee Sensors Journal,* vol. 10, no. 9, pp. 1535-1539, Sep, 2010.

[21] D. Dardari, “Detection and accurate localization of harmonic chipless tags,” *Eurasip Journal on Advances in Signal Processing*, Aug 20, 2015.

[22] S. Lanzisera, D. T. Lin, and K. S. J. Pister, “RF time of flight ranging for wireless sensor network localization,” *Proceedings of the Fourth International Workshop on Intelligent Solutions in Embedded Sysems*, pp. 165-+, 2006.

[23] K. Uchino, “The Development of Piezoelectric Materials and the New Perspective,” *Advanced Piezoelectric Materials: Science and Technology, 2nd Edition*, pp. 1-92, 2017.

1. We assume the detuning of the mechanical mode is zero, $\Delta_{b}=0$. [↑](#footnote-ref-2)
2. Please note that this approach is not exact. To obtain the exact picture, it is necessary to fully solve the equations of motion, as discussed in the previous subsection. Nevertheless, the aim of this subsection is to illustrate the analogy of our systems to the Kuramoto model, representing the connection of comb generation with synchronization. [↑](#footnote-ref-3)
