# Supplementary material for: Passive frequency comb generation at radiofrequency for ranging applications
Source: Nat Commun. 2024 Apr 2;15:2844. doi: 10.1038/s41467-024-46940-2 (PMC10987526; doi:10.1038/s41467-024-46940-2)
Supplement: Supplementary file 3 — Description of Additional Supplementary Files [file 41467_2024_46940_MOESM3_ESM.pdf]

Description of Additional Supplementary Files

File Name: Supplementary Movie 1
Description: A detailed demonstration on the passive generation of frequency comb for the far-field ranging of a drone.
